# Hand-Assisted Laparoscopic Native Nephrectomy for Polycystic Kidney Disease Before Transplantation: A Single-Centre Case Series with a Technique-Stratified Focused Scoping Review

**DOI:** 10.3390/jcm15155856

**Published:** 2026-07-27

**Authors:** Fahim Kanani, Chaya Shwaartz, Mirit Meller, Rotem Horowitz, Tomer Baytner Zamir Yosilevsky, Vladimir Tennak, Ashraf Imam, Abed Khalaileh, Aviad Gravetz, Eviatar Nesher

**Affiliations:** 1 Department of Transplantation, Beilinson Medical Centre, Petah Tikva 4941492, Israel; miritmel8@gmail.com (M.M.); rotem.horowitz.1@gmail.com (R.H.); tomerbaytner@gmail.com (T.B.Z.Y.); vladimirta@clalit.org.il (V.T.); aviadgr@clalit.org.il (A.G.);; 2 Gray Faculty of Medicine, Tel Aviv University, Tel Aviv 6997801, Israel; 3 Department of Transplantation and HPB Surgery, Toronto General Hospital, Toronto, ON M5G 2C4, Canada; chaya.shwaartz@uhn.ca; 4 Organ Transplantation Unit, Department of Surgery, Hadassah Hebrew University Medical Center, Jerusalem 91120, Israel; achrafim@hadassah.org.il (A.I.); hbedk@hadassah.org.il (A.K.); 5 Faculty of Medicine, Hebrew University of Jerusalem, Jerusalem 91120, Israel

**Keywords:** autosomal-dominant polycystic kidney disease, native nephrectomy, hand-assisted laparoscopy, kidney transplantation, post-operative complications, cyst decompression

## Abstract

**Background:** Native nephrectomy is frequently required in autosomal-dominant polycystic kidney disease (ADPKD) before or during transplantation, but the optimal minimally invasive approach is unsettled, and it is unclear whether the pattern of post-operative complications tracks with operative technique. We report a retrospective single-centre experience of hand-assisted laparoscopic (HAL) native nephrectomy and, alongside it, a technique-stratified scoping review of complications. **Methods:** We retrospectively reviewed all consecutive adults undergoing HAL native nephrectomy—with an infra-umbilical midline hand-port and free intraperitoneal cyst rupture—in preparation for transplantation in the period between December 2019 and December 2025. In parallel, we performed a PRISMA-ScR-compliant scoping review (MEDLINE, Embase, Scopus) of minimally invasive ADPKD nephrectomy, with duplicate independent screening and extraction, stratifying complications by operative approach and cyst-decompression method. No quantitative pooling was undertaken. **Results:** Twenty-three patients (mean age 52.8 ± 9.9 years; 87% dialysis-dependent; 78% for transplant preparation) were included. Median operative time was 102 min (IQR 90–122) with minimal blood loss, one transfusion (4%), and no open conversion. Complications occurred in 8/23 (35%) and were bowel-predominant: one small-bowel perforation, two obstructions, and one ileus—three of Clavien–Dindo grade IIIb, all in right-sided nephrectomies and independent of specimen weight. Peri-operative mortality was 1/23 (4%), from a non-technique-related mycotic aortic dissection. Of 481 records screened, 19 studies met inclusion; the cyst-decompression method was reported in only 7 (37%). Bowel events clustered in series using free intraperitoneal cyst rupture or puncture and were largely absent where decompression was contained, a pattern crossing platform boundaries and that is mechanistically consistent with intraperitoneal spillage of cyst contents. **Conclusions:** HAL native nephrectomy is a rapid and feasible means of removing massively enlarged polycystic kidneys before transplantation but, in our experience, carries a bowel-predominant morbidity that clustered in right-sided procedures and was independent of specimen weight. The sparse literature is consistent with—but cannot confirm—a relationship to intraperitoneal cyst spillage rather than to the hand-assisted approach itself. Whether contained cyst decompression can preserve operative efficiency while reducing bowel morbidity is a hypothesis warranting prospective study.

## 1. Introduction

Autosomal-dominant polycystic kidney disease (ADPKD) is the most common inherited nephropathy, affecting roughly 1 in 400 to 1 in 1000 individuals and accounting for an estimated 5–10% of patients reaching kidney failure worldwide [1,2]. It arises predominantly from pathogenic variants in *PKD1* or *PKD2*, which disrupt polycystin signalling and drive progressive cyst expansion, kidney enlargement, and a decline in function [3,4] that culminates in end-stage kidney disease (ESKD) in the majority of affected patients by the sixth decade [5,6]. A substantial proportion of these patients ultimately require kidney replacement therapy, and a large share are dialysis-dependent at the time of surgical and transplant decision-making [7,8].

In a meaningful minority of patients the native kidneys reach massive dimensions, and this burden—through mass effect, recurrent cyst infection, haemorrhage, intractable pain, or simple lack of intra-abdominal domain for an allograft—makes native nephrectomy necessary either before or at the time of transplantation [9,10]. Nephrectomy in this setting is not solely a space-making manoeuvre but also serves to reduce ongoing complications; whether pre-emptive nephrectomy lowers post-transplant morbidity in asymptomatic patients remains contested, and several groups advocate a selective, symptom-driven policy [9,11].

The choice of which side to remove, and whether to remove one kidney or both, is not standardised. Some centres preferentially clear one iliac fossa to accommodate the graft, while the laterality decision is variously driven by which kidney is larger or symptomatic, by the intended side of transplantation, and by anatomical access; the literature reflects genuinely divergent policies rather than a single accepted rule [10,11,12]. This variability in indication, laterality, and timing is one reason the reported outcomes of ADPKD nephrectomy are difficult to compare across series.

That heterogeneity matters because native nephrectomy in ADPKD, while generally safe, is far from innocuous. Contemporary series and a recent consensus synthesis place it in the intermediate-risk category, with overall complication rates on the order of a third of patients and major (Clavien–Dindo grade ≥ III) events in a non-trivial minority [11]. The minimally invasive armamentarium—pure laparoscopic, hand-assisted laparoscopic (HAL), and robotic approaches—has reduced blood loss, transfusion requirement, and length of stay relative to open surgery, but each approach carries a distinct operative footprint, and it is plausible that the *pattern* of complications, not merely their frequency, tracks with the technique and with how the massively enlarged, cyst-laden specimen is manipulated and extracted [13,14,15,16].

Against this background we report a single-centre, seven-year experience of HAL native nephrectomy performed in preparation for transplantation, in which specimen delivery is achieved through an infra-umbilical midline incision using a hand-access device introduced from the outset. We observed a complication profile weighted toward bowel-related events. To place this observation in context, we then undertook a systematically searched, technique-stratified scoping review of the published literature on minimally invasive ADPKD nephrectomy, examining whether complication patterns—in particular bowel and visceral morbidity—correlate with the operative approach and specimen-handling strategy employed. Our aim is descriptive and hypothesis-generating: to characterise our own outcomes honestly and to ask whether the bowel-predominant morbidity we observed is associated with the method of cyst decompression—free intraperitoneal spillage—rather than with the hand-assisted approach itself, a question not previously examined systematically.

## 2. Methods

### 2.1. Study Design and Ethics

This work has two components: a single-centre retrospective case series of hand-assisted laparoscopic (HAL) native nephrectomy for autosomal-dominant polycystic kidney disease (ADPKD), and a systematically searched, technique-stratified scoping review of complications after minimally invasive ADPKD nephrectomy. The cohort study was conducted in accordance with the Declaration of Helsinki and approved by the institutional review board of RMC-0805-23, 4 February 2026; given the retrospective design, the requirement for individual informed consent was waived. The scoping review used only published aggregate data and required no ethical approval.

### 2.2. Scoping Review: Protocol and Reporting

The review was designed and reported in accordance with the Preferred Reporting Items for Systematic Reviews and Meta-Analyses extension for Scoping Reviews (PRISMA-ScR) [17]. A scoping-review framework was chosen deliberately: the anticipated evidence base was heterogeneous in technique, indication, and outcome definition, and dominated by non-randomised observational designs, rendering a pooled quantitative synthesis (meta-analysis) methodologically inappropriate. The review question was framed using the Population–Concept–Context structure—patients with ADPKD undergoing minimally invasive native nephrectomy (Population); operative approach and specimen-handling strategy (Concept); and the pattern of post-operative complications, in particular bowel/visceral versus vascular/access events (Context).

### 2.3. Search Strategy and Information Sources

Three databases were searched: MEDLINE (via PubMed), Embase, and Scopus. The primary strategy combined controlled vocabulary and free-text terms for the disease and the procedure, restricted to minimally invasive approaches. The MEDLINE string was: (“Polycystic Kidney, Autosomal Dominant”[Mesh] OR “Polycystic Kidney Diseases”[Mesh] OR ADPKD[tiab] OR “polycystic kidney”[tiab]) AND (“Nephrectomy”[Mesh] OR nephrectom*[tiab]) AND (laparoscop*[tiab] OR robot*[tiab] OR “hand-assisted”[tiab] OR “hand assisted”[tiab] OR “minimally invasive”[tiab] OR “Laparoscopy”[Mesh] OR “Robotic Surgical Procedures”[Mesh]), with equivalent Emtree and Scopus translations; the complete search strategy for each database is provided in Supplementary Table S1. A broader disease-plus-procedure search and a technique-focused string (incorporating terms for specimen extraction, morcellation, Pfannenstiel, and hilar control) were run as sensitivity checks. Searches were performed in May 2026 and were restricted to records published between January 2016 and May 2026, with no language restriction; reference lists of included studies and relevant reviews were hand-searched to identify additional records.

### 2.4. Study Selection

**Figure 1:**
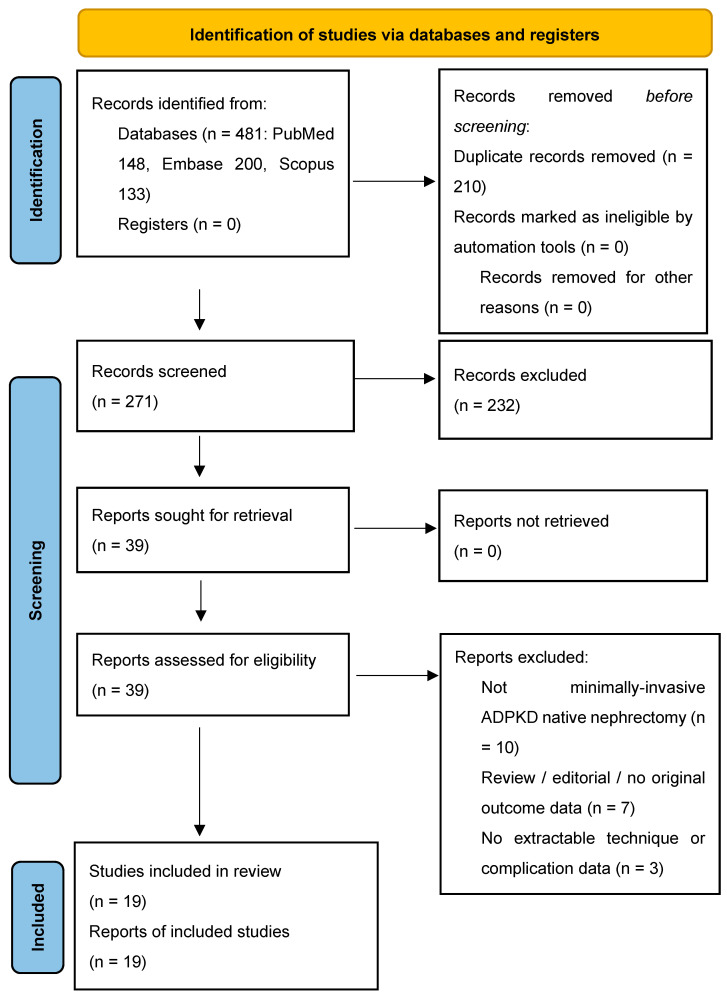
PRISMA-ScR flow diagram of study identification, screening, and inclusion. Records were identified from PubMed (*n* = 148), Embase (*n* = 200), and Scopus (*n* = 133); no automation tools were used for screening or selection.

Records were deduplicated and screened in two stages—title/abstract, then full text—against pre-specified eligibility criteria. Inclusion criteria were: (i) adult patients with ADPKD (Population); (ii) minimally invasive native nephrectomy—pure laparoscopic, hand-assisted, robotic, or retroperitoneal—with or without concurrent transplantation (Concept); and (iii) original clinical reports describing peri-operative complications (Context). Exclusion criteria were: open-only series without a minimally invasive arm; narrative reviews, meta-analyses, and consensus statements (retained for background only); and studies not reporting peri-operative outcomes. No restriction was placed on study design, sample size, or language. Screening and full-text assessment were performed independently by two reviewers (F.K. and A.G.); disagreements were resolved by discussion, and a third senior reviewer (E.N.) adjudicated when consensus could not be reached. The selection process is summarised in a PRISMA-ScR flow diagram (Figure 1).

### 2.5. Data Extraction and Data Items

Data were extracted independently by the same two reviewers into a piloted standardised form and cross-checked. Extracted items comprised: first author and year; study design, country, and period; number of patients and kidneys and the indication mix; operative approach (open, pure laparoscopic, HAL, or robotic) and transperitoneal versus retroperitoneal route; vascular-control strategy (hilum-first versus stepwise); specimen extraction site and—recorded separately—the cyst-decompression method (intact, in-bag aspiration, extracorporeal aspiration, intraperitoneal rupture, or morcellation); kidney weight or volume; operative time, estimated blood loss, transfusion, and conversion to open surgery; complications classified by type (bowel/visceral, vascular/access, wound/infectious, medical, or other) and, where reported, by Clavien–Dindo grade [18]; length of stay; and mortality. Where a datum was not reported it was recorded as “not reported” rather than inferred.

### 2.6. Methodological Appraisal

Each included study was assigned a level of evidence using the Oxford Centre for Evidence-Based Medicine (OCEBM) 2011 hierarchy [19], and its methodological quality was appraised narratively using the Joanna Briggs Institute critical-appraisal tools appropriate to case series and cohort designs [20]. Because almost all eligible studies were small, retrospective, and unadjusted for the principal confounders (kidney size, indication, laterality, bilaterality, dialysis status, and surgeon experience), appraisal focused on selection, completeness of outcome ascertainment, and reporting of complication definitions; these limitations are carried explicitly into the interpretation.

### 2.7. Single-Centre Cohort: Patients and Data

We reviewed all consecutive adults (≥18 years) with ADPKD who underwent HAL native nephrectomy in preparation for, or at the time of, kidney transplantation at our institution between December 2019 and December 2025, with no exclusion by laterality, comorbidity, or dialysis status. Demographic, clinical, operative, and outcome data were retrieved from the electronic medical record: age, sex, dialysis status, comorbidities, operative side and laterality, indication, operative time, estimated blood loss, intra-operative transfusion, specimen weight, pre-operative and first post-operative-day (POD-1) serum creatinine and haemoglobin, post-operative complications, 30-day readmission, length of stay, mortality, and interval to subsequent transplantation. All procedures were performed by one of three consultant transplant surgeons, each with more than ten years of post-fellowship experience and an individual annual volume of at least five such nephrectomies; the hand-assisted, hilum-first protocol described below has been the department’s standardised approach since 2019, ensuring a uniform operative technique across the cohort. Follow-up extended from the date of surgery to May 2026.

### 2.8. Surgical Technique

The technique follows the hilum-first minimally invasive approach previously described by our collaborating group [21], with a single deliberate modification in access and extraction. The patient is positioned in the lateral decubitus position on the contralateral side with a table break to open the costophrenic angle. In contrast to the purely laparoscopic index technique—in which the specimen is retrieved through a separate Pfannenstiel incision at the end of the procedure—an infra-umbilical midline incision is made at the outset and a GelPoint hand-access device is placed, so that the operating surgeon’s non-dominant hand assists throughout the procedure and the same incision later serves as the extraction site. Working ports are placed under vision and pneumoperitoneum maintained at 15 mmHg. The colon is mobilised medially and Gerota’s fascia is opened to expose the kidney. Consistent with the hilum-first principle, the renal hilum is approached early: after dissection of the inferior renal border, the renal artery and vein are divided together at the hilum with a vascular stapler before extensive mobilisation or cyst decompression, and the ureter is subsequently clipped and divided. The kidney is enucleated from the perinephric fat with adrenal preservation. To reduce the volume of the massively enlarged specimen and permit delivery through the hand-port incision, large cysts are ruptured and decompressed by hand within the peritoneal cavity, and the collapsed kidney is then delivered intact through the infra-umbilical hand-port incision. The fascia and skin are closed in layers; drains are placed selectively. This intraperitoneal manual cyst decompression, together with the hand-assisted transperitoneal mobilisation, distinguishes our approach from the purely laparoscopic index technique, in which cysts are aspirated in a contained fashion within the retrieval bag before extraction [21]. This intraperitoneal manual decompression was adopted from the outset for pragmatic reasons: it is rapid, requires no additional retrieval device, and allows a massively enlarged specimen to be collapsed and delivered through a single hand-port incision, contributing to the short operative times observed. The technique remained unchanged throughout the study period. Notably, no specific measures to contain cyst contents—such as in-bag decompression, controlled aspiration, or peritoneal irrigation after rupture—were routinely employed; this omission is precisely the modifiable step that our findings bring into question.

### 2.9. Outcomes and Definitions

The primary descriptive outcomes were the occurrence and type of post-operative complications, classified as bowel/visceral, vascular/access, wound/infectious, medical, or other, and graded by the Clavien–Dindo classification [18]; major complications were defined as grade ≥ III. Secondary outcomes were operative time, estimated blood loss (EBL was recorded qualitatively as minimal in all cases and was not routinely quantified in millilitres; this is noted as a limitation of the retrospective record), transfusion, specimen weight, POD-1 renal function and haemoglobin, length of stay, 30-day readmission, peri-operative mortality, and time to transplantation. Peri-operative mortality was defined as death from any cause within 30 days of nephrectomy or during the same hospital admission (Clavien–Dindo grade V); deaths occurring later, during the transplant-preparation follow-up interval, were recorded and reported separately and were not counted in the peri-operative surgical-morbidity analysis.

### 2.10. Statistical Analysis

Analyses were descriptive. Continuous variables are presented as median (interquartile range) or mean ± standard deviation, and categorical variables as counts and percentages. Given the modest sample size, the cohort analysis was not powered for formal hypothesis testing, and no inferential comparison is presented for the single-arm data; the review data are summarised descriptively and stratified by technique, without pooling.

## 3. Results

### 3.1. Part 1—Single-Centre HAL Cohort

#### 3.1.1. Patients and Baseline Characteristics

Between December 2019 and December 2025 23 consecutive adults with autosomal-dominant polycystic kidney disease (ADPKD) underwent hand-assisted laparoscopic (HAL) native nephrectomy in preparation for, or at the time of, kidney transplantation. The mean age was 52.8 ± 9.9 years, and 15 of 23 patients (65%) were male. Twenty of 23 patients (87%) were dialysis-dependent at the time of operation, and none had a functioning prior kidney transplant. Indications were not mutually exclusive; where more than one was present, the principal indication was used for classification, so that category counts exceed the cohort size. The median pre-operative serum creatinine was 6.83 mg/dL (IQR 4.45–9.20 mg/dL) and the median pre-operative haemoglobin was 11 g/dL (IQR 10–13). Hypertension was present in 17 of 23 patients (74%), ischaemic heart disease in 7 of 23 (30%), and diabetes mellitus in 4 of 23 (17%). The principal indication was preparation for transplantation in 18 of 23 patients (78%), followed by flank or abdominal pain in 5 of 23 (22%) and recurrent urinary tract infection in 1 of 23 (4%). Operative laterality was markedly right-dominant: 16 procedures were right-sided, 4 left-sided, and 3 bilateral. Baseline characteristics are presented in Table 1.

#### 3.1.2. Intra-Operative and Post-Operative Outcomes

The median operative time was 102 min (IQR 90–122). Estimated blood loss was minimal in all cases, one patient (4%) received an intra-operative packed-red-cell transfusion, and no procedure was converted to open surgery. The median specimen weight was 1440 g (IQR 1090–2282). The median first post-operative-day (POD-1) creatinine was 8.2 mg/dL (IQR 6.2–8.9) and the median POD-1 haemoglobin was 11 g/dL (IQR 10–12), corresponding to a median haemoglobin fall of 1 g/dL (IQR 0–1). The median length of stay was 5 days (IQR 4–8). No malignancy was identified on pathological examination. Intra-operative and post-operative outcomes are summarised in Table 2.

#### 3.1.3. Complications

A post-operative complication occurred in 8 of 23 patients (35%). The profile was weighted toward bowel-related events: one small-bowel perforation requiring re-laparotomy with resection and anastomosis (Clavien–Dindo grade IIIb), two small-bowel obstructions (grade IIIb, one of which required intensive-care admission), and one ileus (grade I). The remaining events were two suspected infections, one post-operative cholangitis (all grade II), and one fatal mycotic aortic dissection (Clavien–Dindo grade V), occurring 12 days after nephrectomy during the index admission. Peri-operative mortality was 1 of 23 (4%); this was an infective vascular catastrophe in a comorbid, vasculopathic dialysis patient and was not attributable to the nephrectomy technique. Thirty-day readmission was recorded in 1 of 21 patients (5%). All four bowel-related events occurred in right-sided nephrectomies—4 of 16 right-sided cases, versus none in the four left-sided and none in the three bilateral procedures. The three grade IIIb events were associated with prolonged hospitalisation (17–26 days). Neither specimen weight nor operative time distinguished affected from unaffected patients: median specimen weight was 1330 g (range 1090–4380) in the bowel-complication group versus ≈1470 g (720–3500) without, and median operative time was 100 versus 102 min. The single heaviest specimen (4380 g) sustained an obstruction, but the remaining three events occurred at or below the cohort median specimen weight, so the pattern is one of laterality rather than specimen bulk. Individual events are detailed in Table 3A,B, and a descriptive comparison in Table 3C. For context, overall complication rates reported across the comparative literature are summarised in Supplementary Table S4; these are presented descriptively and were not pooled.

### 3.2. Part 2: Scoping Review

#### 3.2.1. Included Studies

Of 481 records identified (PubMed 148, Embase 200, Scopus 133), 19 original clinical studies met the inclusion criteria (Figure 1, Supplementary Table S2). They were published between 2020 and 2026 and comprised 8 retrospective comparative cohorts, 7 single-arm case series, and 4 case reports; no randomised or prospective comparative study was identified, and all corresponded to OCEBM level 3 (*n* = 10) or level 4 (*n* = 9). Cohort size ranged from single-patient reports to comparative series of 52–148 patients. The operative approach was hand-assisted or lap-assisted laparoscopic in 7 studies, robotic in 5, pure laparoscopic in 3, retroperitoneal laparoscopic in 1, and predominantly open in 2; the remaining comparative cohorts contributed more than one approach arm. Vascular control was hilum-first in five studies and stepwise in four, and was unstated in the remainder. The specimen-extraction site was reported inconsistently, and the cyst-decompression method—the pre-specified variable of interest—was described in only 7 of 19 studies (37%).

#### 3.2.2. Complications by Technique Stratum

Complication profiles differed in type across strata (Table 4). The seven hand-assisted/lap-assisted studies reported bowel-related events (ileus, obstruction, or enterotomy) together with vascular/access events, and two of them identified delayed recovery of bowel function as the principal morbidity of the procedure [15,16]. The three pure-laparoscopic and five robotic studies reported few bowel events and proportionally more vascular/access and wound complications; a single robotic series was the exception, reporting two cases of post-operative ileus [22]. The open arms carried the most severe events: the robotic-versus-open comparison recorded visceral injury in 2 of 15 open patients (combined hepatic, splenic, diaphragmatic, and common-bile-duct injury requiring secondary procedures) and none of 17 robotic patients [23], and ADPKD nephrectomy was associated with a higher burden of post-operative hypotension, anaemia, and transfusion than non-ADPKD nephrectomy [24].

#### 3.2.3. Study-Level Complication Counts

Bowel-related events were reported in five verified studies: enterotomy or bowel injury (Abrol and Prieto [15], *n* = 1; Huynh [12], *n* = 1 colonic injury), bowel obstruction (Abrol [16], *n* = 1 reoperation), and ileus (Gurung [22], *n* = 2; Huynh [12] open arm, *n* = 1; Shweiki [21], *n* = 1). No bowel event was reported in the robotic series of Masterson [25], Vianna [26], or Spaggiari [27], in the hand-assisted series of Chalklin [28], or in the case reports of Shaheen [29], Penbegul [30], or Nazario-Perez [31]. Access-related vascular events were confined to dialysis-dependent cohorts (AV-fistula thrombosis or revision in Huynh [12], Collini [14], and Shweiki [21]). Throughout, we distinguish the absence of *reported* bowel complications from the *confirmed* absence of such events: in most series bowel outcomes were simply not stated, and a nil count cannot be assumed. Only studies explicitly reporting bowel outcomes are treated as informative for that variable. Counts were not pooled, given heterogeneity in design, case-mix, approach, and outcome definitions. One heterogeneous open/mixed-approach cohort [32] is retained as a verified included study but, as it does not report complications in a bowel-stratified form, it is presented descriptively in Supplementary Table S2 and is not entered into the study-level bowel-complication count (Supplementary Table S3).

#### 3.2.4. Cyst-Decompression Method and Bowel Complications

Among the seven studies that reported cyst decompression (Table 5), 3 used free intraperitoneal rupture or puncture, 3 used contained handling (in-bag fragmentation or deliberate avoidance of puncture), and 1 used retroperitoneal puncture. Bowel events occurred in 2 of the 3 intraperitoneal-decompression series [15,22], the third not reporting bowel outcomes [13]. The three contained-handling series were essentially bowel-free, the only event being a single ileus in one [21,25,28]. One robotic series stated explicitly that intentional cyst puncture may spill infectious and irritating contents capable of producing ileus and a chemical-peritonitis-like reaction [25]. In the single retroperitoneal series, cyst puncture was performed outside the peritoneal cavity and gastrointestinal morbidity was limited to diarrhoea, with no ileus or obstruction [33].

## 4. Discussion

In this single-centre series of HAL native nephrectomy performed in preparation for kidney transplantation, the salient finding is a complication profile weighted toward the gastrointestinal tract. Half of all recorded complications, and all four of the events graded Clavien–Dindo IIIb, were bowel-related: one small-bowel perforation, two small-bowel obstructions, and one ileus [18]. These events dominated the morbidity of an otherwise expeditious operation performed with minimal blood loss and no conversion to open surgery. They were also clinically consequential, accounting for the longest hospital stays in the series (17–26 days) and clustering in right-sided procedures. This is not a trivial pattern, and it frames the central question of the present work: whether the bowel-predominant morbidity we observed is an idiosyncrasy of our cohort or a reproducible consequence of how a massively enlarged, cyst-laden kidney is decompressed and delivered.

### 4.1. A Mechanistic Reading: Cyst Spillage Rather than the Hand

The most instructive result of the accompanying scoping review is not a difference in complication *rate* but a difference in complication *type* that appears to track with a single, often-overlooked operative step—the method of cyst decompression (Table 5). Reporting of this variable was sparse: it was described in only a minority of the nineteen included studies, which is itself an important observation about the completeness of the surgical literature. Where it was reported, however, the direction was consistent. Series that ruptured or punctured cysts freely within the peritoneal cavity recorded bowel events, whereas series that kept decompression contained—within a retrieval bag, or by deliberately avoiding puncture altogether—did not. The pivotal observation is that this pattern crossed the boundary between operative platforms. A robotic series that punctured large cysts intraperitoneally to gain working space reported post-operative ileus [22], while a robotic series that explicitly avoided cyst puncture reported none and stated the rationale directly: intentional puncture may spill infectious and irritating contents capable of producing ileus and a chemical peritonitis-like reaction [25]. A hand-assisted series using contained in-bag fragmentation likewise recorded no bowel events [28], as did the purely laparoscopic reference technique employing in-bag rupture with extracorporeal suction [21]. Conversely, the hand-assisted series that reduced specimen volume by blunt intraperitoneal rupture identified delayed recovery of bowel function as its principal morbidity [15,16].

Our own practice sits unambiguously on the intraperitoneal side of this divide: cysts are ruptured freely within the peritoneal cavity to collapse the specimen before delivery through the hand-port incision. Read against Table 5, our bowel-predominant profile appears concordant with, rather than anomalous to, the pattern seen in the few other series that report intraperitoneal cyst spillage—a consistency we interpret cautiously, given how rarely the variable is reported. We are careful not to overstate this. The mechanism is explicitly articulated in only one source [25], the supporting series are small and retrospective, and cyst handling is unreported in most of the literature, so the association is at best suggestive. Nonetheless, the convergence of an explicit pathophysiological rationale, a consistent direction across the studies that do report the variable, and our own concordant experience is more consistent with intraperitoneal cyst spillage than with the hand-assisted approach per se as the associated factor, though this remains a hypothesis rather than a demonstrated cause. These observations suggest that intraperitoneal cyst handling, rather than the hand-assisted approach itself, deserves further investigation as a potentially modifiable operative step; we advance this as a hypothesis rather than as an established mechanism.

### 4.2. Complication Patterns by Technique

Viewed across strata (Table 4), the broader literature is consistent with this interpretation. Open series carried the most severe visceral and vascular events—organ laceration, bowel perforation, and haemorrhage requiring transfusion—reflecting the exposure demanded by giant kidneys, and dialysis-dependent ADPKD patients undergoing nephrectomy shows a correspondingly high burden of post-operative hypotension, anaemia, and transfusion [24]. A contemporary robotic-versus-open comparison reported visceral injuries only in the open arm with two patients sustaining combined hepatic, diaphragmatic, splenic, and common-bile-duct injuries requiring secondary procedures [23]. Purely laparoscopic and robotic series concentrated their morbidity in vascular-access and wound events, several of which the authors attributed to the patients’ dialysis dependence rather than to the operation itself [13,21]. Hand-assisted and lap-assisted series occupied an intermediate position, combining access-related events with the bowel morbidity discussed above [12,15,16,28]. A retroperitoneal laparoscopic series is illuminating precisely because it breaks the expected link: cysts were punctured, but within the retroperitoneal plane rather than the peritoneal cavity, and the reported gastrointestinal morbidity was limited to diarrhoea, with no ileus or obstruction [33]. Compartment, not merely puncture, appears to matter.

### 4.3. Operative Time, Laterality, and the Trade-Off

The counterpoint to our bowel morbidity is speed. Our median operative time of 102 min was substantially shorter than that reported for totally laparoscopic hilar nephrectomy [21], robotic nephrectomy [22,25,26,27], and pure laparoscopic series [13], and approached the operative times of open surgery [33]. Shorter operative duration is not a cosmetic advantage in this population: reduced anaesthetic exposure, and lower cardiopulmonary and thromboembolic burden, are meaningful in dialysis-dependent patients with a high prevalence of ischaemic heart disease, in whom operative physiological stress is poorly tolerated. The same open intraperitoneal access and manual cyst rupture that accelerate specimen reduction and delivery are, however, the very steps implicated in bowel morbidity. The possible trade-off—a faster operation at the price of a higher bowel-complication burden—is one reading of our data, but it is confounded on several axes that we cannot adjust for in a cohort of this size: right-sided predominance, specimen weight, comorbidity and dialysis dependence, unilateral-versus-bilateral scope, and a plausible learning-curve effect across a seven-year single-centre series. Our cases were predominantly unilateral, whereas several comparators performed longer bilateral or simultaneous transplant procedures [15,16,26,27], so technique and operative scope in particular cannot be disentangled.

Two further sources of heterogeneity deserve comment because they complicate any cross-series comparison. First, laterality: Our cohort was markedly right-dominant (16 of 23), and all four bowel events occurred in right-sided nephrectomies, whereas specimen weight did not distinguish affected patients—arguing against specimen mass alone as the explanation. Whether the right-sided clustering reflects the anatomical proximity of the duodenum, hepatic flexure, and right colon to the operative and cyst-decompression field, or simply the right-predominance of the cohort, cannot be determined from these numbers, and we resist over-interpreting a four-event denominator. The choice of side and of unilateral versus bilateral nephrectomy is itself unstandardised across the literature: some groups clear the intended graft fossa, others are guided by the larger or symptomatic kidney, and consensus favours a selective, unilateral, minimally invasive approach unless a specific indication dictates otherwise [11]. Second, indication: The proportion operated purely to create space for transplantation, as opposed to for pain, infection, or haemorrhage, varies widely between series and shapes both case selection and the threshold for intervention [9,11,12]. These differences in laterality, extent, and indication are a principal reason that complication rates cannot be pooled across studies and must be read descriptively.

### 4.4. Limitations

Our study has important limitations. The cohort is small, retrospective, and single-centre, and no adjustment for kidney size, indication, dialysis status, or bilaterality is possible; the bowel-complication signal, while clinically coherent, rests on four events. The single peri-operative death was a fatal mycotic aortic dissection on the twelfth post-operative day of the index admission—a catastrophic infective vascular event in a comorbid, vasculopathic dialysis patient, not plausibly attributable to the nephrectomy technique, and we have not counted it as procedure-specific morbidity. The scoping review, though systematically searched and independently screened, comprises exclusively level 3–4 evidence with heterogeneous and frequently incomplete outcome reporting; the key mechanistic variable, cyst decompression, was unreported in most studies, and one included cohort could not be entered into the technique-stratified complication count because it did not report bowel-specific events separately, although its inclusion status and bibliographic details are confirmed. Quantitative synthesis was neither performed nor appropriate. Our findings are consequently hypothesis-generating and should be interpreted as a pattern to be tested, not a causal relationship established.

## 5. Conclusions

Hand-assisted laparoscopic native nephrectomy with intraperitoneal cyst decompression and midline extraction is a feasible and rapid means of removing massively enlarged polycystic kidneys before transplantation, but in our experience it carries a bowel-predominant morbidity. The wider literature, sparse as it is on this point, is compatible with the hypothesis that this morbidity relates less to the hand-assisted approach itself than to free intraperitoneal spillage of cyst contents—a potentially modifiable step, but one that our data can only signal, not establish. Whether contained decompression can preserve the operative efficiency of the hand-assisted technique while reducing bowel morbidity is a question our data raises but cannot answer, and one that merits prospective, technique-explicit study.

## Figures and Tables

**Table 1:** Baseline and pre-operative characteristics of the HAL cohort (*n* = 23).

Variable	Value
Age, mean ± SD, years	52.8 ± 9.9
Male sex, *n* (%)	15/23 (65%)
Body mass index(BMI), median (range), kg/m^2^	27 (25–30)
Side—left:right:bilateral	4:16:3
Pre-operative dialysis, *n* (%)	20/23 (87%)
Prior kidney transplant, *n* (%)	0/23 (0%)
Pre-operative creatinine, median (IQR), mg/dL	6.83 (4.45–9.20)
Pre-operative haemoglobin, median (IQR), g/dL	11 (10–13)
Hypertension, *n* (%)	17/23 (74%)
Diabetes mellitus, *n* (%)	4/23 (17%)
Ischaemic heart disease, *n* (%)	7/23 (30%)
Previous abdominal surgery, *n* (%) *	3/23 (13%)
Indication—transplant preparation, *n* (%)	18/23 (78%)
Indication—flank/abdominal pain, *n* (%)	5/23 (22%)
Indication—recurrent UTI, *n* (%)	1/23 (4%)

* Previous abdominal surgery defined as prior intra-abdominal or pelvic operation; inguinal hernia repair not included.

**Table 2:** Intra-operative and post-operative outcomes (*n* = 23).

Variable	Value
Operative time, median (IQR), min	102 (90–122)
Estimated blood loss	Minimal *
Intra-operative transfusion (PRBC), *n* (%)	1/23 (4%)
Conversion to open, *n* (%)	0/23 (0%)
Specimen weight, median (IQR), g	1440 (1090–2282)
POD-1 creatinine, median (IQR), mg/dL	8.2 (6.2–8.9)
POD-1 haemoglobin, median (IQR), g/dL	11 (10–12)
Haemoglobin drop, median (IQR), g/dL	1 (0–1)
Length of stay, median (IQR), days	5 (4–8)
Malignancy on pathology, *n* (%)	0/23 (0%)
Any post-operative complication, *n* (%)	8/23 (35%)
30-day readmission, *n* (%)	1/21 (5%)
Peri-operative mortality (Clavien–Dindo V), *n* (%)	1/23 (4%)

* EBL not quantified in mL; recorded qualitatively.

**Table 3:** (**A**) Post-operative complications, management, and outcome (HAL cohort). (**B**) Per-event detail of the four bowel complications (all right-sided, unilateral nephrectomies). (**C**) Descriptive comparison of patients with and without bowel complications (no inferential testing).

(**A**)								
**Complication**			**Management**			**Clavien–Dindo**	**LOS, Days**	**Outcome**
Small-bowel perforation			Re-laparotomy, resection + anastomosis			IIIb	26	Survived
Small-bowel obstruction			Re-operation; ICU (3 d)			IIIb	25	Survived
Small-bowel obstruction			Surgical management			IIIb	17	Survived
Ileus			Conservative; readmitted with fever			I	8	Resolved
Cholangitis			Antibiotics/drainage			II	4	Survived
Suspected infection			Empirical antibiotics			II	4	Survived
Suspected infection			Empirical antibiotics			II	9	Survived
Aortic dissection			Peri-operative death			V	12	Died †
(**B**)								
**Case**	**Side**	**Specimen wt (g)**	**Op time (min)**	**Complication**	**CD Grade**	**Management**	**LOS (d)**	**Final Outcome**
1	Right	1220	89	Ileus	I	Conservative management	8	Resolved; not yet transplanted at last follow-up
2	Right	1440	122	Small-bowel perforation	IIIb	Re-laparotomy; small-bowel resection and anastomosis	26	Recovered; subsequently transplanted
3	Right	4380	99	Small-bowel obstruction	IIIb	Reoperation; intra-operative transfusion; ICU admission ‡	25	Recovered; subsequently transplanted
4	Right	1090	101	Small-bowel obstruction	IIIb	Reoperation	17	Recovered; subsequently transplanted
(**C**)								
**Variable**				**Bowel Complication (*n* = 4)**		**No Bowel Complication (*n* = 19)**		
Right-sided				4 (100%)		12 (63%)		
Left-sided				0		4 (21%)		
Bilateral				0		3 (16%)		
Median specimen weight, g (range) §				1330 (1090–4380)		≈1470 (720–3500)		
Median operative time, min				100		102		
Median length of stay, days (range)				21 (8–26)		5 (3–12)		

† The single peri-operative death (Clavien–Dindo grade V) was a mycotic aortic dissection on post-operative day 12 of the index admission, an infective vascular event not attributable to the nephrectomy technique. HAL, hand-assisted laparoscopic; LOS, length of stay; ‡ ICU admission attributed to Case 3. Management of the grade IIIb events reflects operative intervention under general anaesthesia, as required by the Clavien–Dindo grade. Time to onset of complication was not routinely recorded and is therefore not tabulated. CD, Clavien–Dindo; LOS, length of stay; ICU, intensive care unit. § Specimen-weight row restricted to unilateral cases with a recorded weight; the three bilateral procedures and two cases without a recorded weight are excluded from this row only. All other rows use the full cohort (*n* = 23). Values are *n* (%) or median (range). Given the sample size, no statistical comparison was performed; differences are presented descriptively.

**Table 4:** Complications by technique stratum.

Technique Stratum	Studies	Dominant Bowel/Visceral Pattern	Dominant Vascular/Access Pattern	Typical Cyst Decompression
Hand-assisted/lap-assisted	7	Ileus, bowel obstruction, enterotomy—reported more frequently	AV-fistula thrombosis (dialysis-linked)	Intraperitoneal rupture (Abrol) vs. contained in-bag (Chalklin)
Pure laparoscopic	3	Few; none in most reports	Access-related (shunt occlusion)	Intraperitoneal disruption (Thomas)/NR
Robotic	5	None when cysts not punctured (Masterson); ileus when punctured intraperitoneally (Gurung)	Rare	Contained vs. intraperitoneal puncture
Open	~5 arms	Severe: ileal perforation, organ laceration	Major bleeding/transfusion	NR/open-field puncture

**Table 5:** Cyst-decompression method and bowel complications (the mechanistic variable).

Study	Cyst Handling	Compartment	Bowel Event?	Explicit Ileus Link in Text
Masterson 2023 [25]	Avoids intentional puncture	Contained	None	Yes—states puncture may cause spillage → ileus/chemical peritonitis
Chalklin 2022 [28]	In-bag splitting	Contained	None	None
Shweiki 2025 [21]	In-bag rupture + external suction	Contained	None (1 ileus) *	None
Abrol & Prieto 2020 [15]	Blunt finger rupture	Intraperitoneal	Yes (bowel injury + ileus)	None
Gurung 2021 [22]	Puncture of large cysts	Intraperitoneal	Yes (2 ileus)	None
Thomas 2023 [13]	Disruption + aspiration	Intraperitoneal	NR	None
Lyu 2024 [33]	Cyst puncture	Retroperitoneal	None (diarrhoea only)	None
Belinson (index)	Free intraperitoneal rupture	Intraperitoneal	Yes (4 bowel events)	—
Others (Huynh [12], Collini [14], Shin [34], Shaheen [29], Penbegul [30], Nazario-Perez [31], Vianna [26], Spaggiari [27], Castaneda [23], Chan [24])	Not reported	—	—	—

* Shweiki’s single ileus is noted for completeness; its cyst handling is contained. No denotes a study that explicitly reported no bowel complications; ‘NR: Not reported’ denotes a study that did not address bowel outcomes. The two are not equivalent and are not pooled.

## Data Availability

The data supporting the findings of this study are available from the corresponding author upon reasonable request.

## Supplementary Materials

The following supporting information can be downloaded at https://www.mdpi.com/article/10.3390/jcm15155856/s1, Table S1: Database-specific search strategies for the scoping review; Table S2: Characteristics of the 19 included studies; Table S3: Study-level complication counts (verified studies; bowel-relevant columns shown); Table S4: Overall complication rates reported across the comparative series included in the scoping review.

## References

[1] Cornec-Le Gall E., Alam A., Perrone R.D. (2019). Autosomal dominant polycystic kidney disease. Lancet.

[2] Torres V.E., Harris P.C., Pirson Y. (2007). Autosomal dominant polycystic kidney disease. Lancet.

[3] Bergmann C., Guay-Woodford L.M., Harris P.C., Horie S., Peters D.J.M., Torres V.E. (2018). Polycystic kidney disease. Nat. Rev. Dis. Primers.

[4] Harris P.C., Torres V.E. (2009). Polycystic kidney disease. Annu. Rev. Med..

[5] Gabow P.A. (1993). Autosomal dominant polycystic kidney disease. N. Engl. J. Med..

[6] Wilson P.D. (2004). Polycystic kidney disease. N. Engl. J. Med..

[7] Spithoven E.M., Kramer A., Meijer E., Orskov B., Wanner C., Abad J.M., Aresté N., de la Torre R.A., Caskey F., Couchoud C. (2014). Renal replacement therapy for autosomal dominant polycystic kidney disease (ADPKD) in Europe. Nephrol. Dial. Transplant..

[8] Akoh J.A. (2015). Current management of autosomal dominant polycystic kidney disease. World J. Nephrol..

[9] Fuller T.F., Brennan T.V., Feng S., Kang S.M., Stock P.G., Freise C.E. (2005). End-stage polycystic kidney disease: Indications and timing of native nephrectomy relative to kidney transplantation. J. Urol..

[10] Tillou X., Timsit M.O., Sallusto F., Culty T., Verhoest G., Doerfler A., Thuret R., Kleinclauss F. (2016). Polycystic kidney disease and kidney transplantation. Prog. Urol..

[11] Geertsema P., Gansevoort R.T., Arici M., Capasso G., Cornec-Le-Gall E., Furlano M., Fuster D.G., Galletti F., Gómez-Dos-Santos V., Perez-Gomez M.V. (2025). Nephrectomy in autosomal dominant polycystic kidney disease: A consensus statement of the ERA Genes & Kidney Working Group. Nephrol. Dial. Transplant..

[12] Huynh N., Yoon P., Hort A., Yao J., Lee T., Yuen L., Laurence J.M., Pleass H. (2022). Utilizing the same incision for staged renal transplant in patients with polycystic kidney disease requiring hand-assisted laparoscopic nephrectomy. ANZ J. Surg..

[13] Thomas M.N., Datta R.R., Wahba R., Buchner D., Chiapponi C., Kurschat C., Grundmann F., Urbanski A., Tolksdorf S., Müller R. (2023). Introduction of laparoscopic nephrectomy for autosomal dominant polycystic kidney disease as the standard procedure. Langenbecks Arch. Surg..

[14] Collini A., Benigni R., Ruggieri G., Carmellini M. (2021). Laparoscopic nephrectomy for massive kidneys in polycystic kidney disease. JSLS.

[15] Abrol N., Prieto M. (2020). Simultaneous Hand-assisted Laparoscopic Bilateral Native Nephrectomy and Kidney Transplantation for Patients With Large Polycystic Kidneys. Urology.

[16] Abrol N., Bentall A., Torres V.E., Prieto M. (2021). Simultaneous bilateral laparoscopic nephrectomy with kidney transplantation in patients with ESRD due to ADPKD: A single-center experience. Am. J. Transplant..

[17] Tricco A.C., Lillie E., Zarin W., O’Brien K.K., Colquhoun H., Levac D., Moher D., Peters M.D.J., Horsley T., Weeks L. (2018). PRISMA extension for scoping reviews (PRISMA-ScR): Checklist and explanation. Ann. Intern. Med..

[18] Dindo D., Demartines N., Clavien P.A. (2004). Classification of surgical complications: A new proposal with evaluation in a cohort of 6336 patients. Ann. Surg..

[19] (2011). OCEBM Levels of Evidence Working Group. The Oxford 2011 Levels of Evidence. Oxford Centre for Evidence-Based Medicine. https://www.cebm.ox.ac.uk/resources/levels-of-evidence.

[20] Munn Z., Barker T.H., Moola S., Tufanaru C., Stern C., McArthur A., Stephenson M., Aromataris E. (2020). Methodological quality of case series studies: An introduction to the JBI critical appraisal tool. JBI Evid. Synth..

[21] Shweiki A., Khalayleh H., Rivin M., Shabaneh S., Khalaileh A., Imam A. (2025). Minimally invasive nephrectomy for the management of polycystic kidney disease: The hilum-first technique. J. Clin. Med..

[22] Gurung P.M.S., Frye T.P., Rashid H.H., Joseph J.V., Wu G. (2021). Robot-assisted synchronous bilateral nephrectomy for autosomal dominant polycystic kidney disease: A stepwise description of technique. Urology.

[23] Castaneda P., Masterson J., Naser-Tavakolian A., Kim I., Najjar R., Gupta A. (2025). Surgical outcomes of robotic bilateral nephrectomy compared to open surgery in adult polycystic kidney disease. World J. Urol..

[24] Chan J.E.Z., Olakkengil K.S., Bhattacharjya S., Olakkengil S.A. (2025). Autosomal Dominant Polycystic Kidney Disease Patients Requiring Nephrectomy: Characteristics and Surgical Considerations. ANZ J. Surg..

[25] Masterson J.M., Zhao H., Taich L., Naser-Tavakolian A., Johnson H., Najjar R., Kim I.K., Gupta A. (2023). Robotic bilateral nephrectomy for large polycystic kidney disease. BJUI Compass.

[26] Vianna R., Morsi M., Munoz-Abraham A.S., Guerra G., Ciancio G. (2026). Robotic-assisted simultaneous bilateral native nephrectomy and living donor kidney transplantation. J. Surg. Res..

[27] Spaggiari M., Almario J., Aguiluz G., Furian L., Bartlett S., Di Cocco P., Tzvetanov I.G., Benedetti E., Giulianotti P.C. (2022). Simultaneous robotic-assisted bilateral native nephrectomy and kidney transplantation for ADPKD in recipients with high body mass index: Report of 2 cases. Transplant. Proc..

[28] Chalklin C.G., Harris C.R., Riddick A.C. (2022). Hand-assisted laparoscopic bilateral native nephrectomy for autosomal dominant polycystic kidney disease. Int. J. Urol..

[29] Shaheen M.F., Aljehaiman F., Altheaby A. (2024). Semi-simultaneous hand-assisted laparoscopic bilateral nephrectomy and kidney transplantation from the same incision in ADPKD: First case report in Saudi Arabia. J. Surg. Case Rep..

[30] Penbegul N. (2020). Simultaneous laparoscopic bilateral nephrectomy in polycystic kidney disease using same trochars placed in the midline. Int. Braz. J. Urol..

[31] Nazario-Perez M.Z., Torres-Arroyo A., Brito-Sanchez R.A., Ruiz-Deya G. (2025). Minimally invasive management of horseshoe kidney with polycystic kidney disease: A case report. Int. J. Surg. Case Rep..

[32] Navratil P., Chalupnik J., Merkl T., Spacek J., Matyskova Kubisova M., Safranek R., Novak I., Pacovsky J., Navratil P., Gunka I. (2025). Native nephrectomy in patients with autosomal dominant polycystic kidney disease in kidney transplant program: Long-term single-center experience. Int. Urol. Nephrol..

[33] Lyu X., Du C.-K., Zhu Y.-C. (2024). Comparison between retroperitoneal laparoscopic nephrectomy and traditional open nephrectomy to treat polycystic kidney disease before kidney transplantation. Urol. J..

[34] Shin H., Choi N.-K. (2024). Incidental renal cell carcinoma post bilateral nephrectomy in autosomal dominant polycystic kidney disease. World J. Clin. Cases.

