# Supplementary material for: Hand-Assisted Laparoscopic Native Nephrectomy for Polycystic Kidney Disease Before Transplantation: A Single-Centre Case Series with a Technique-Stratified Focused Scoping Review
Source: J Clin Med. 2026 Jul 27;15(15):5856. doi: 10.3390/jcm15155856 (PMC13467154; doi:10.3390/jcm15155856)
Supplement: Supplementary file 1 [file jcm-15-05856-s001.zip › jcm-4445527-supplementary.pdf]

Supplementary Table S1. Database-specific search strategies for the scoping review.

**Database (interface)**	**Search strategy**	**Records**
MEDLINE (via PubMed)	("Polycystic Kidney, Autosomal Dominant"[Mesh] OR "Polycystic Kidney Diseases"[Mesh] OR ADPKD[tiab] OR "polycystic kidney"[tiab]) AND ("Nephrectomy"[Mesh] OR nephrectom*[tiab]) AND (laparoscop*[tiab] OR robot*[tiab] OR "hand-assisted"[tiab] OR "hand assisted"[tiab] OR "minimally invasive"[tiab] OR "Laparoscopy"[Mesh] OR "Robotic Surgical Procedures"[Mesh]) Filters: 2016/01/01:2026/05/31[dp]	148
Embase (via Emtree / Elsevier)	('autosomal dominant polycystic kidney disease'/exp OR 'polycystic kidney disease'/exp OR adpkd:ti,ab OR 'polycystic kidney':ti,ab) AND ('nephrectomy'/exp OR nephrectom*:ti,ab) AND ('laparoscopy'/exp OR 'laparoscopic surgery'/exp OR 'robot assisted surgery'/exp OR 'minimally invasive surgery'/exp OR laparoscop*:ti,ab OR robot*:ti,ab OR 'hand assisted':ti,ab OR 'hand-assisted':ti,ab OR 'minimally invasive':ti,ab) AND [2016-2026]/py	200
Scopus	TITLE-ABS-KEY("autosomal dominant polycystic kidney" OR "polycystic kidney disease" OR ADPKD OR "polycystic kidney") AND TITLE-ABS-KEY(nephrectomy OR nephrectom*) AND TITLE-ABS-KEY(laparoscop* OR robot* OR "hand-assisted" OR "hand assisted" OR "minimally invasive") AND PUBYEAR > 2015 AND PUBYEAR < 2027	133
**Total records identified**		**481**

All searches were performed in May 2026 and restricted to records published between January 2016 and May 2026, with no language restriction.

The Embase and Scopus strategies are database-appropriate translations of the MEDLINE strategy, with controlled vocabulary (Emtree) and field syntax adapted to each interface. Truncation is denoted by an asterisk (*); [Mesh] = Medical Subject Heading; [tiab] = title/abstract; :ti,ab = title/abstract (Embase); /exp = Emtree term with explosion; TITLE-ABS-KEY = title, abstract, and keywords (Scopus).

In addition to the primary strategy, a broader disease-plus-procedure search and a technique-focused string (incorporating terms for specimen extraction, morcellation, Pfannenstiel, and hilar control) were run as sensitivity checks, and the reference lists of included studies and relevant reviews were hand-searched. No automation tools were used at any stage of screening or selection.

Supplementary Table S2. **Characteristics of the 19 included studies.**

Author (year)	Country	Design	n (kidneys)	Approach	Vascular control	Extraction	Cyst decompression	Op time (min)	LoE
Abrol & Prieto 2020	USA	Retrospective cohort	52 (104)	HAL	Hilum-first	Pfannenstiel hand-port	Intraperitoneal blunt rupture	310 (med)	3
Abrol 2021	USA	Retrospective case-control	148 (BNKT 51 / KT-alone 97)	HAL	Hilum-first	Pfannenstiel	Intraperitoneal rupture	NR	3
Huynh 2022	Australia	Retrospective comparative	35 (HALN 22/Lap 8/Open 5)	Mixed	Stepwise	Rutherford-Morrison	NR	NR	3
Collini 2021	Italy	Retrospective comparative	35 (48 nx; HALN 26/open 22)	HAL vs open	Hilum-first	Pfannenstiel/subumbilical	NR	180 vs 90 (uni)	3
Chalklin 2022	UK	Retrospective case series	10 (20)	HAL	NR	Periumbilical midline hand-port	Contained (in-bag splitting)	282 (mean)	4
Shin 2024	South Korea	Retrospective cohort	19 (38; open 8/HALN 11)	Mixed	NR	NR	NR	120 (mean)	3
Shaheen 2024	Saudi Arabia	Case report	1 (2)	HAL	NR	Pfannenstiel→port	NR	NR	4
Thomas 2023	Germany	Retrospective comparative	21 (24; Lap 12/open 12)	Pure lap vs open	Stepwise	Suprapubic piecemeal	Intraperitoneal disruption + aspiration	158 vs 107	3
Penbegul 2020	Turkey	Video case report	1 (2)	Pure lap	NR	Midline below umbilicus	NR	NR	4
Nazario-Perez 2025	USA (PR)	Case report	1 (2, horseshoe)	3D lap	Stepwise (segmental)	Extended port; retrieval bag	NR	135	4
Masterson 2023	USA	Retrospective case series	14 (28)	Robotic	Hilum-first	Extended midline port	Contained — avoids puncture	299 (med)	4
Gurung 2021	USA	Retrospective case series	7 (14)	Robotic	Uncertain	Lower-midline port; bag	Intraperitoneal puncture (>2.5 L)	388 (med)	4
Vianna 2026	USA	Retrospective case series	4 (8)	Robotic	NR	Pfannenstiel + GelPort	NR	462.5 (med)	4
Spaggiari 2022	USA	Case report / 2-case	2 (4)	Robotic	NR	GelPort	NR	395 (mean)	4
Peris Castaneda 2025	USA	Retrospective comparative	32 (RNx 17/ONx 15)	Robotic vs open	NR	NR	NR	NR (not increased)	3
Lyu 2024	China	Retrospective comparative	57 (RL 23/open 34)	Retroperitoneal lap vs open	Stepwise	Entrapment bag 10 cm / 20 cm flank	Retroperitoneal puncture (not intraperitoneal)	201 vs 113 (mean)	3

Author (year)	Country	Design	n (kidneys)	Approach	Vascular control	Extraction	Cyst decompression	Op time (min)	LoE
Chan 2025	Australia	Retrospective comparative	ADPKD vs non-ADPKD	Mixed (mostly open)	NR	NR	NR	NR	3
Navratil 2023 †	Czech Republic	Retrospective cohort	89	Open	NR	Subcostal/midline	NR	NR	3
Shweiki 2025	Israel	Retrospective case series	16 (17)	Pure lap / robotic	Hilum-first	Pfannenstiel/prior-Tx	Contained — in-bag rupture + external suction	159.6 (mean)	4

Supplementary Table S3. **Study-level complication counts (verified studies; bowel-relevant columns shown).**

Author (year)	Approach	Ileus	Obstruction	Enterotomy/perforation	Other visceral	AVF/access	Reoperation	Mortality
Abrol & Prieto 2020	HAL	3 (readmission)	–	1 (bowel injury)	0	0	NR	0
Abrol 2021	HAL	delayed bowel recovery (major morbidity)	1 (adhesiolysis)	NR	0	NR	1	0
Huynh 2022	Mixed	1 (open arm)	0	1 (HALN colonic injury)	0	1 (HALN)	NR	NR
Collini 2021	HAL vs open	0	0	0	0	1 (AVF revision)	NR	1 (open, pneumonia)
Chalklin 2022	HAL	0	0	0	0	0	1 (exploration)	NR
Thomas 2023	Lap vs open	NR	NR	NR	NR	NR	1/arm (CD ≥III)	NR
Masterson 2023	Robotic	0	0	0	0	0	NR	0
Gurung 2021	Robotic	2 (CD I)	0	0	0	0	0	0
Vianna 2026	Robotic	0	0	0	0	0	0	0
Spaggiari 2022	Robotic	0	0	0	0	0	0	0
Castaneda 2025	Robotic vs open	NR	NR	NR	RNx 0 / ONx 2	NR	ONx 2	NR
Lyu 2024	Retroperit. lap vs open	0 (diarrhoea only)	NR	0	0	NR	NR	0
Nazario-Perez 2025	3D lap	0	0	0	0	0	0	0
Shweiki 2025	Lap/robotic	1	0	0	0	2 (AVF)	0	0

Case reports with no events — Shaheen, Penbegul — are omitted here; the Navrátil cohort is retained in Supplementary Table S2 but is not bowel-stratified and is therefore not counted here.

Supplementary Table S4 **Overall complication rates reported across the comparative series included in the scoping review.**

**Study (approach)**	**n (kidneys)**	**Overall complication rate**	**Bowel-specific events**
Present series (HAL, intraperitoneal rupture)	23	35% (30% excl. non-technique death)	4 (17%): perforation ×1, obstruction ×2, ileus ×1
Abrol & Prieto 2020 (HAL, intraperitoneal)	52	NR	bowel injury + delayed bowel recovery
Abrol 2021 (lap, bilateral+KT)	NR	NR	obstruction (1 reoperation)
Chalklin 2022 (HAL, contained in-bag)	NR	NR	0
Masterson 2023 (robotic, contained)	14	NR	0
Gurung 2021 (robotic, intraperitoneal puncture)	NR	NR	ileus ×2
Thomas 2023 (pure lap)	NR	NR	NR
Lyu 2024 (retroperitoneal)	NR	NR	0 (diarrhoea only)

Rates are presented descriptively and were not pooled, given heterogeneity in study design, case-mix, operative approach, and definitions of post-operative complications. Single-patient case reports are not included, as complication rates are not meaningfully calculable from them.
